# Prediction and injury risk based on movement patterns and flexibility in a 6-month prospective study among physically active adults

**DOI:** 10.7717/peerj.11399

**Published:** 2021-05-18

**Authors:** Dawid Koźlenia, Jarosław Domaradzki

**Affiliations:** Faculty of Physical Education and Sport, Biostructure Unit, University School of Physical Education in Wrocław, Wrocław, Dolnośląskie, Polska

**Keywords:** Movement patterns, Functional Movement Screen, FMS, Flexibility, Injury, Injury risk, Injury prediction, Physical activity, Young adults

## Abstract

**Background:**

Physical activity has many health benefits but also carries a risk of injury. Some universal factors are connected with an increased risk regardless of the type of sport. Identifying these factors may help predict injuries and aid in their prevention.

**Aim:**

The aim of this study is to determine the level of injury risk and the accuracy of injury prediction during a prospective 6-month period based on the quality of movement patterns and level of flexibility among average physically active young adults.

**Material and Methods:**

A group of 123 young, physically active adults were recruited for this study. The International Physical Activity Questionnaire (IPAQ) was used to determine their level of physical activity. The author’s own Injury History Questionnaire (IHQ) was used to retrospectively collect injury data from the 12 months before the study and prospectively collect data during the six month observation period. The Functional Movement Screen (FMS) test was conducted to assess the movement patterns quality and a sit-and-reach test was conducted to measure lower back and hamstrings flexibility.

**Results:**

Low-quaility movement patterns (14≥ FMS) increased the injury risk level sevenfold. A level of flexibility reduced by one cm increased the risk of injury by 6%. Previous injuries also increased the risk of injury reccurence 6.4 times. Predicting injury occurrence based on the quality of movement patterns allows for an accuracy of 73%, whereas flexibility allows for a 41% accuracy. The simultaneous use of these two factors did not improve injury prediction accuracy.

**Conclusion:**

The risk of an injury increases with low-quality movement patterns, a low level of flexibility, and previous injuries. Preventative strategies should include shaping high-quality movement patterns, the right level of flexibility, and the full healing of injuries before resuming activities. The quality of movement patterns is an accurate predictor of injury risk, but lower back and hamstrings flexibility is not a reliable predictor of injury.

## Introduction

Physical activity has many health benefits but can lead to injury (Meeuwisse et al., 2007). Many studies confirm the prevalence of injuries that occur during physical activity among ordinary people around the world (Złotkowska et al., 2015; Marar et al., 2012; Cai et al., 2018). Injuries may be caused by incorrect warm-up, excessive physical load (Ristolainen et al., 2014), previous injuries (Giroto et al., 2017), poor quality movement patterns (Kiesel, Plisky & Voight, 2007; Garrison et al., 2015), and a low level of motor fitness, especially flexibility (De la Motte et al., 2019a; De la Motte et al., 2019b). Injuries may be predicted by identifying the determinants of injuries and preventive measures may reduce the risk of injury (Emery & Pasanen, 2019).

Cook et al. (2010) indicated that is possible to predict injury based on the quality of movement patterns assessed using the Functional Movement Screen (FMS) test (Cook et al., 2010). Numerous studies have indicated that low FMS scores (14 ≥ FMS) are associated with a higher number of injuries (Kiesel, PJ & Voight, 2007; Chorba et al., 2010; Chimera et al., 2015). An elevenfold increase in the risk of injury was found in people with low-quality movement patterns (14 ≥ FMS) (Kiesel, PJ & Voight, 2007). Bunn, Rodrigues & BezerradaSilva (2019) conducted a systematic review with meta-analysis to show that subjects with low FMS scores are more likely to suffer injury.

However, observations of various durations were conducted, providing ambiguous results. Kiesel, PJ & Voight (2007) and Duke, Martin & Gaul (2017) indicated that it is possible to accurately predict injuries when assessing the quality of movement patterns in studies spanning 4.5 months and 8 months, respectively. However, studies conducted by Słodownik, Ogonowska-Słodownik & Morgulec-Adamowicz (2018) and De la Motte et al. (2019a); De la Motte et al. (2019b) suggest that it is not possible to predict an injury within 6 months of taking an FMS test. Most studies were conducted in groups of athletes and soldiers. There are no similar studies on the average physically-active population.

Existing discrepancies indicate the need to consider other comorbid factors associated with injuries. They include the level of motor skills, especially flexibility (De la Motte et al., 2019a; De la Motte et al., 2019b). Appropriate flexibility of soft tissues allows for better absorption of forces acting on them during physical activity (Safran, Seaber & Garrett, 1989). Lower levels of flexiblity put tissues at risk for damage by these forces (McHugh et al., 1999). This is evidenced by studies conducted by Bradley & Portas (2007) and Gabbe et al. (2005) among football players, which indicated that a low level of flexibility is related to an increase in injuries.

There are no studies on the determinants of injuries that have simultaneously captured the quality of the movement patterns and flexibility, although it has been suggested that higher quality movement patterns are associated with a higher level of flexibility (Chimera et al., 2017; Yildiz, Pinar & Gelen, 2019). However, both, movement patterns and flixibility are factors predispose to injury (Kiesel, PJ & Voight, 2007; Garrison et al., 2015; De la Motte et al., 2019a; De la Motte et al., 2019b). Further study of simultaneous use movement patterns quality and flexibility should allow for a more accurate prediction of injury. Few authors have taken this approach in the literature on FMS and motor fitness assessment. Lisman et al. (2013) indicated that combining the FMS score with a 3-mile run time allowed a more accurate prediction of an injury’s occurrence. Other observations provided by Kodesh et al. (2015) indicate that there is no relationship between FMS assessment, running time at various distances, and injury.

The literature lacks data for estimating the risk of injury using the two factors as a movement patterns quality and flexibility. Available studies show inconsistent results and cannot be not applied to a large population of average people undertaking various physical activities. Lehr et al. (2013) indicated that a more comprehensive assessment may be effective in predicting injury.

Therefore, the aim of this study is to determine the level of injury risk and the accuracy of injury prediction during a prospective 6-month period based on the quality of movement patterns and level of flexibility among average physically active young adults. Obtained results should identified factors increase the risk of injury and predictive possibilities, allowing to select appropriate methods to reduce the risk of injury during physical activity.

## Materials & Methods

### Characteristics of the study sample

The study sample consisted of 123 young, physically active people with a mean age 23.45 ± 1.12. Detailed characteristics of the studied groups are found in Table 1. Participants were physically active volunteers, did not have experience in professional sports, and did not have injuries six weeks before starting the study measurements. They were recruited from students of a Faculty of Physical Education & Sport. All subjects were required to sign a written consent before participating in this study. They were informed in detail about the purpose, type, research methodology, and participation conditions. Participants were allowed to withdraw from research at any time without giving any reason.

The research was carried out in the Biokinetics Research Laboratory of the Academy of Physical Education, which has a Quality Management System Certificate PN-EN ISO 9001: 2009 (Certificate Reg. No. PW-48606-10E). The Senate Research Ethics Committee approved the research at the University of Physical Education in Wrocław following the ethical requirements for human experiments under the Helsinki Declaration (consent number 16/2018).

### Measurements

We used a height gauge model 764 (SECA manufactured, Hamburg, Germany. Quality control number C-2070) to measure height and weight. We calculated the body mass index (BMI) based on the obtained values of height and weight:

\begin{eqnarray*}\mathrm{BMI}= \frac{\text{body mass}[\mathrm{kg}]}{\text{body height}[{\mathrm{m}}^{2}]} . \end{eqnarray*}

Physical activity level was determined using the The International Physical Activity Questionnaire (IPAQ). It is a validated and reliable tool widely used in researches. (Biernat, Stupnicki & Gajewski, 2007). This questionnaire let to measure and assess the physical activity level of young, middle-aged adults, and nonprofessional sports people. This tool required self-reported information about the average time spent on physical activity (minutes per week). The obtained data allow to calculate the number of Metabolic Equivalents of Task - MET, based on which is possible to determine whether a person is physically active. MET for walk =3.3; MET for moderate physical activity =4.0; MET for vigorous physical activity =8.0. The general MET for a person is counted using the formula: MET (intensity of physical activity) × number of days × number of minutes = MET-minutes/week. For this study below-mentioned inclusion criteria were adopted for physically active people:

**Table 1:** Characteristics of research group. FMS Overall and sit-and-reach test results.

Variable	mean	SD
Body mass [kg]	73,10	13,99
Body height [m]	1,75	0,10
BMI [kg/m2]	23,82	3,05
Physical activity level I [MET]	3770,69	2457,31
Physical activity level II [MET]	4309,39	2652,10
FMS – Overall	14,95	2,33
Sit-and-reach test [cm]	12,62	6,98

- 3 or more days of vigorous exercise, for a total of at least 1500 MET-min/week,

- 7 or more days of any combination of exercise (walking, moderate or vigorous exercise) exceeding 3000 MET-min/week.

The Injury History Questionnaire (IHQ), was used to collect the injury data of the study participants. The IHQ consists a simple question concern about the number of injuries in a clearly specified period. The survey was conducted in a supervised manner. The researcher was at the respondents’ disposal all time to answer any possible uncertainties and doubts while filling in the questionnaire.

The IHQ reliability verification was assessed 7 days after participants took the IHQ; the survey was repeated among a randomly selected group of 56 people. The IHQ reliability was determined used alpha-Cronbacha coefficient which at level 0.836 indicated high reliability of IHQ (Aron, Aron & Coups, 2012).

The injury was defined in this study as the occurrence of complaints during physical activity, which resulted in pain and discomfort in the locomotor system, causing temporary limitations or a complete inability to continue physical activity (Widuchowski & Widuchowski, 2005).

The FMS test was used to assess the quality of movement patterns, which included a battery of seven movement tasks: Deep Squat (DS); Hurdle Step (HS); In-line Lunge (IN-L); Shoulder Mobility (SM); Active Straight Leg Raise (ASLR); Trunk Stability Push-up (TSPU); and Rotary Stability (RS). The FMS test assesses the functional state of the locomotor system through the prism of motor control, mobility, and stability. Individual motor tasks were rated on a scale of zero to three according to clear guidelines described for each test (Cook et al., 2010). Three points were awarded when a subject performed the movement entirely correctly. Two points were awarded when the subject made a move with visible compensation. One point was awarded when the subject could not perform the given movement task correctly. Zero points were awarded when the subject reported pain during a movement test, regardless of the quality of the displayed movement pattern. The FMS test also included three, additional tasks which were not included in the overall assessment to identify potential pain symptoms. Each movement task was performed up to three times. In the case of unilateral trials, the lower grade was taken into account for the overall score. Thus, the possible maximum score was 21 points. The risk of injury increased significantly at 14 or fewer points (Kiesel, PJ & Voight, 2007). The literature indicates that there is a possible different cut-off point value (Letafatkar et al., 2014; Dorrel et al., 2018). Calculating the cut-off point for the study group can reduce the interpretation error. The division cut-off will be the value of the cut-off point determined by the Receiver Operating Characteristic (ROC) method.

Flexibility of the lower back and hamstrings was determined using a sit-and-reach test on a table measuring 35 cm long, 45 cm wide, and 32 cm high and another tabletop, which was 55 cm long. The tabletop protruded 15 cm above the sidewall against which the subject placed their feet. A measuring tape was placed on the tabletop, parallel to the long axis, measuring zero to 50 cm. An indicator was loosely applied perpendicularly, and was used to move the subject with his hands during the test. The subject sat down with their legs straight at the knee joints and both feet flat against the table’s sidewall. The subject was asked to bend forward, trying to move the ruler on the table as far as possible along the scale while maintaining the extension in the knee joints. The better result of each trial was considered. The measurement was performed with an accuracy of 0.5 cm.

### Study design

(1) A survey along with the IPAQ and IHQ questionnaires to document injuries sustained in the last 12 months (retrospectively) and measure physical activty level.

• IHQ reliability verification was assessed 7 days after participants took the IHQ; the IHQ was repeated among a randomly selected group of 56 people (28 men and 28 women).

(2) Assessment of the participants’ quality of movement patterns, level of flexibility, and morphological structure.

• The verification of the FMS test’s reliability and conformity of the FMS assessment were assessed during the FMS test, in which cameras were used to record the movements of 56 randomly-selected people (28 men and 28 women). The recordings were used to visually verify the FMS assessment. FMS assessment repeatability was assessed using 30 randomly selected people (15 men and 15 women) 7 days after the first study.

(3) 6-month observation period –started immediately after the examination with the FMS and flexibility tests.

(4) Re-examination with the IPAQ and AAHU questionnaires for injuries sustained during 6 months of follow-up (prospectively towards the FMS and flexibility measurments).

### Statistics

The reliability of the FMS test was assessed using the Interclass Correlation Coefficient (ICC). Choosen model 2.1 (intrarater reliability) and ICC 3.1 (test-retest reliability). The level of reliability of the ICC value is evaluated on the following scale: weak: 0.00-−0.50; moderate: 0.50-−0.75; good: 0.75-−0.90; excellent: above 0.90 (Koo & My, 2016). Cronbach’s alpha coefficient was calculated to determine the reliability of the IHQ survey. The value of the coefficient for a reliable survey tool should be at least 0.60 (Aron, Aron & Coups, 2012). In the random selection for reliability tests, the sampling frame was a list of respondents by surname in alphabetical order. The Shapiro–Wilk test was used to test the normality of the distributions of the analyzed variables. The means and standard deviations were calculated for data meeting the normal distribution or the median, and standard errors were calculatd for data that were not normally distributed. The ROC method was used to divide the participants into groups with high and low FMS and flexibility injury risk, according to the established cut-off point. The curve makes it possible to determine the optimal point of data division according to the assumed criterion. Logistic regression was used to calculate the odds ratio of an injury occurrence. Injury prediction accurate was based on selected factors and was calculated using injury data collected during the 6-month follow-up period. We used the following formula (Kołakowska, 2018):

\begin{eqnarray*}\mathrm{accuracy = } \frac{\mathrm{true~ positives~ scores + true~ negatives~ scores}}{\mathrm{all~ scores}} \times 100\text{%}. \end{eqnarray*}

The χ2 test compared the prediction accuracy of an injury’s occurrence or absence. The analyses were performed in groups selected by the quality of movement patterns and flexibility ROC curve.

A significance level of α = 0.05 was adopted for all statistical tests and is marked in bold in the tables. We used Statistica v13.0 by Statsoft Polska for statistical analysis.

## Results

The Cronbach’s alpha coefficient for IHQ was 0.836. This indicates very good reliability and repeatability of the survey (Aron, Aron & Coups, 2012). Functional Movement Screen - Compliance of self-assessment (video recordings) ICC (2.1): FMS overall assessment - 0.96; Repeatability of self-assessment (re-assessment) ICC (3.1): FMS overall assessment - 0.95.

Table 1 shows descriptive statistics of the studied group.

Table 2 presents the incidence of injuries in the retrospective and prospective periods in the research group.

**Figure 1:**
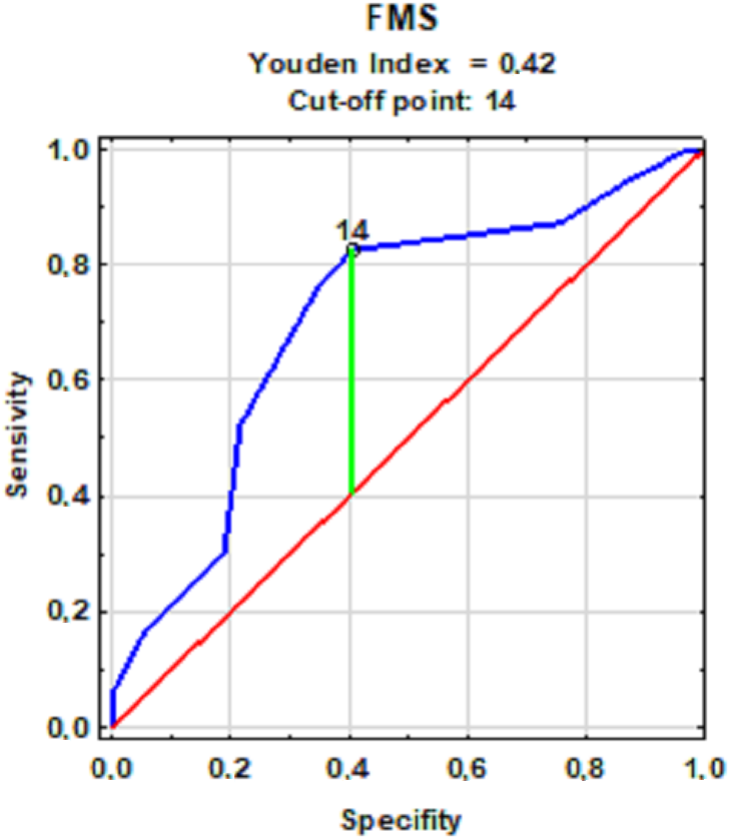
The cut-off point for groups with low and high-quality movement patterns with a frequency of injuries.

The FMS score cut-off value was 14 points (Fig. 1). An increased frequency of injuries in the retrospective period, 12 months prior to the study was observed in subjects with a result equal to or lower than the FMS test cut-off point. On this basis, the participants were divided into groups of high-quality movement patterns (14 <FMS) in whom injuries occurred less frequently and people with low-quality movement patterns (14 ≥FMS) in whom injuries occurred more often.

**Table 2:** The incidence of injuries in retrospective and prospective periods.

Period	Injury	N	%
Retrospective	No	87	70,73
	Yes	36	29,26
Prospective	No	98	79,67
	Yes	25	20,32

**Figure 2:**
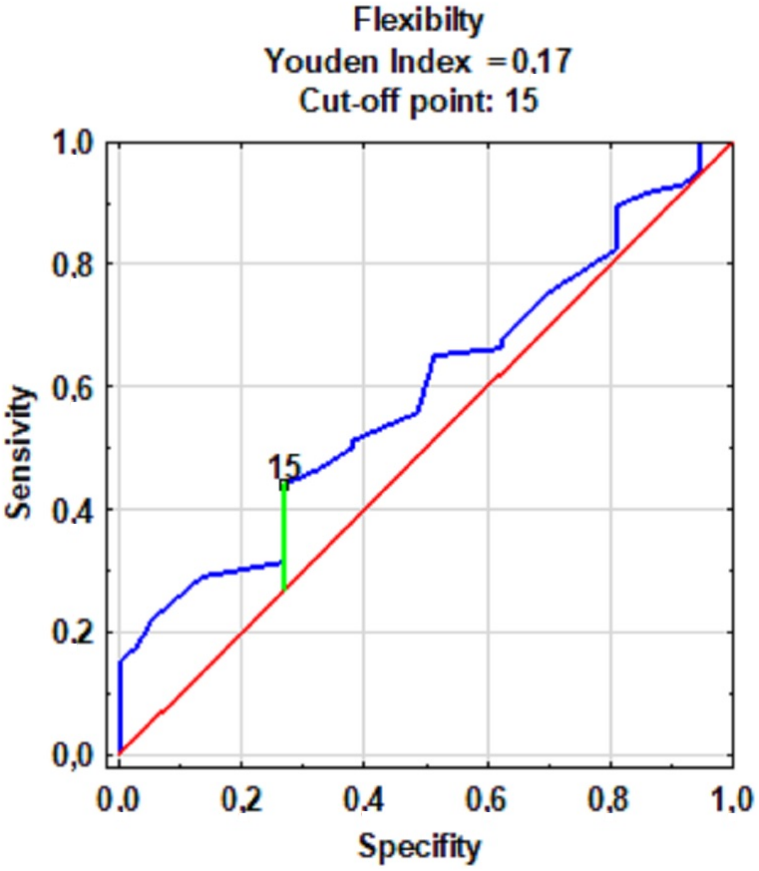
The cut-off point for groups with low and high levels of flexibility with the frequency of injuries.

The cut-off point value for the level of flexibility lower back and hamstrings was 15 cm (Fig. 2). An increased frequency of injuries in the retrospective period 12 months prior to the start of the study was observed in subjects with a score equal to or below the flexibility cut-off point. On this basis, the participants were divided into groups with a high (15 cm <Sit-and-reach score) and low (15 cm ≥Sit-and-reach score) level of flexibility.

The quality of movement patterns and quantitative and qualitative (dichotomous) flexibility and previous injury state (injury retrospectively) were included in analyzing the risk of injury. Table 3 shows the logistic regression models. Subjects with low-quality movement patterns (14 ≥FMS) were nearly seven times more likely to experience an injury than people with high-quality patterns. This model is statistically significant. A one unit (1 cm) decrease in flexibility may increase the risk of injury by 6%. Additionaly, subjects who suffered injuries in the retrospective period were 6.4 times more likely to suffer injury again prospectively. However, there was no reliability to the interactions between the above-mentioned factors. The quantified flexibility factor was used to show that the logistic regression model is statistically significant and indicates an injury’s predictive potential. The two-factor predictive models are not statistically significant. Neither model demonstrates predictive reliability.

**Table 3:** Injury risk prediction models in terms of quality of movement patterns and qualitative and quantitative flexibility using retrospective injuries.

Factor	Interaction	Rate	SE	Stat. Wald	GU +	GU -	*P*	Odds ratio	CI OR −95%	CI OR +95%
movement patterns (14 ≥ FMS)	–	1.942	0.435	19.975	1.090	2.794	**≤0.0001**	6.974	2.976	16.346
quantitative flexibility	–	−0.062	0.030	4.276	−0.121	−0.003	**0.0387**	0.940	0.886	0.997
qualitative flexibility (15 cm ≤)	–	0.734	0.412	3.172	−0.074	1.542	0.0743	2.083	0.929	4.673
injury retrospectively	–	1.866	0.471	15.680	0.942	2.789	**0.000**	6.460	2.566	16.266
movement patterns - quantitative flexibility	0.96	0.008	0.088	0.007	−0.165	0.180	0.9321	1.008	0.848	1.197
movement patterns - qualitative flexibility (15 cm ≤)	0.93	0.153	1.184	0.017	−2.167	2.473	0.8972	1.165	0.115	11.859
movement patterns-injury retrospectively	0.28	0.997	1.070	0.869	−1.100	3.095	0.351	2.711	0.333	22.078
quantitative flexibility-injury retrospectively	0.89	0.048	0.106	0.202	−0.160	0.255	0.653	1.049	0.852	1.291
qualitative flexibility (≥15 cm)-injury retrospectively	0.42	0.762	1.320	0.333	−1.825	3.349	0.564	2.142	0.161	28.475

The prediction of an injury’s occurrence was verified with the quality factors of movement patterns and flexibility of lower back and hamstrings. The division of the subjects was determined based on ROC curve models for the quality of movement patterns (Fig. 1) and flexibility (qualitative approach) (Fig. 2). Injury was expected for low-quality movement patterns (14 ≥FMS) and low levels of flexibility quantitative (15 cm ≥Sit-and-reach score). However, no injuries were expected with high-quality movement patterns and a high level of flexibility. The accuracy of the predictions was verified with the actual state (occurrence of an injury or its absence) and the predicted state (the expected injury or its absence) using the data on injuries sustained during the observation.

Table 4 compares the groups for injury occurence in terms of the predicted state and the actual state using the ROC curve model for the quality of movement patterns from data prospectively collected during the 6-month observation period.

**Table 4:** Accuracy of the prediction model for an injury with the quality of movement patterns and flexibility.

Factor	Quality of movement patterns			Flexibility		
Injury Occurrence - Predicted state	Injury Occurrence - The actual state		*χ* ^2^	Injury Occurrence - The actual state		*χ* ^2^
	Yes	No	*df* = 1*χ*2 = 15.99≤*p* = 0.0001	Yes	No	*df* = 1*χ*2 = 0.80*p* = 0.3704
Yes	17	25		12	68	
No	8	73		4	39	
Prediction accuracy	73.18% (CI 95 [0.64–0.80])			41,14% (CI 95 [0.31–0.49])		

The accuracy of predictions based on the quality of movement patterns was 73.18%. The χ2 statistic value indicates that at the significance level of 0.0001, we can reject the hypothesis about the independence of the predicted and actual state with the occurrence of an injury. However, in the case of lower back and hamstrings flexibillity, the accuracy of predictions was 41.14%. The χ2 statistic value indicates that at the significance level of 0.6708, we cannot reject the hypothesis about the predicted state’s independence and the actual state with the injury occurrence.

## Discussion

The risk of injury is ubiquitous, regardless of the type and level of physical activity undertaken (Złotkowska et al., 2015; Marar et al., 2012; Cai et al., 2018). Our study sought to determine the risk of injuries among young, physically active adults and the assessment of the accuracy of injury prediction was based on the quality of movement patterns and the level of flexibility. The ability to accurantely predict injury allows participants to take preventive measures to avoid injury (Emery & Pasanen, 2019). Garrison et al. (2015) demonstrated the accuracy of the prediction of injury based on the FMS assessment. A significant level for injury risk was observed among people with an overall FMS score below the cut-off point. The risk of injury was nearly six times higher in those with lower quality movement patterns than in those with a higher quality of movement patterns. In the case of people with previous injuries, their risk increased 15 times. This result is almost two times higher than our research, where the risk of injury in the case of low-quality movement patterns (14 ≥ FMS) was seven times higher. However, research by Garrison et al. (2015) was conducted on athletes. Similarly, in a study of rugby players Duke, Martin & Gaul (2017) indicated that those characterized by a lower quality of movement patterns were more than ten times more likely to be injured. The risk of injury was correlated with longer breaks from training due to injuries sustained during the season. A study by Chorba et al. (2010) conducted on 38 athletes showed a 4-fold increase in injury risk with poor movement patterns than in people with good quality movement patterns. This model accurantely predicted injuries among 69% of the examined women over a period of a year. Kiesel, PJ & Voight (2007) found an 11-fold risk of injury based on their assessment over 4.5 month, during which the accuracy of the prediction of injury was indicated at 51%. Our research indicated a 73% accuracy of prediction during the observation period. Some authors indicate lack of accuracy prediction based on the FMS assessment. De la Motte et al. (2019a); De la Motte et al. (2019b) used several functional tests, including FMS, but did not indicate the possibility of predicting injury in a large group of over 1,600 soldiers during the 180-day observation period. Słodownik, Ogonowska-Słodownik & Morgulec-Adamowicz (2018) examined injuries among handball players retrospectively (12 months) with the FMS assessment and 6 months after (observation period), and did not show simple relationships between the quality of movement patterns and injuries. This suggests that other factors related to the risk of injury should be considered. Literature reports indicate a strong relationship between flexibility and injury (De la Motte et al., 2019a; De la Motte et al., 2019b). Witvrouw et al. (2004) showed that footballers with lower muscle flexibility, manifested by weaker ranges of motion in the joints, were more likely to be injured than those with standard values. In the following years, these results were confirmed by Bradley & Portas (2007), also indicating that greater flexibility protects against injuries.

The predictive model in our research on the flexibility factor showed that a decrease in its value by one cm was associated with a 6% increase in injury risk. Witvrouw et al. (2003) indicated that preseason measurements of the level of flexibility among soccer players may be a valuable diagnostic tool in predicting injury. Prospective studies of young male and female runners showed associations between less flexibility and overload injuries (Yagi, Muneta & Sekiya, 2013).

However, as Arnason *et al.*(2008) showed that flexibility alone is not enough to prevent injury. A holistic approach is essential, including appropriate training of other motor skills. This explains the lack of accuracy of the predictive model in our research, where 41.14% of injuries were correctly predicted based on flexibility. Similarly, Van Doormaal et al. (2017) did not show the possibility of predicting the occurrence of an injury in the next 12 months on this basis when examining the flexibility of 450 amateur soccer players.

However knowing the determinants of injuries and the possibility of their diagnosis, there are many coexisting factors that often intertwine, which may ultimately determine the possibility of predicting an injury (Moore et al., 2019). Despite the reports (Chimera et al., 2017; Yildiz, Pinar & Gelen, 2019) indicating the relationship between the quality of movement patterns and flexibility, few researchers have considered the common influence of these factors on an injury.

We found that participants injured in retrospective period were 6.4 times more likely to suffer injury again in a prospective period. Unhealed injuries may cause constant pain, become reinjured and worsen, and may cause further injuries (Meeuwisse et al., 2007; Swenson et al., 2009).

The recognition of the connection between the quality of movement patterns and flexibility of the lower back and hamstrings could enable a more accurate prediction of an injury’s occurrence. However, until now, attempts have been made to predict injuries based on the quality of movement patterns using other motor skills. Lisman et al. (2013) indicated that a slower time in the 3-mile run combined with lower quality patterns was more strongly associated with injuries. People who had weaker scores in both areas were four times more likely to be injured. Kodesh et al. (2015) made a similar attempt to assess the determinants of injuries. However, their research showed the relationship between injuries and endurance without indicating the relationship between FMS scores and injuries in a group of 145 female soldiers. We attempted to define the determinants of injuries taking into account quality of movement patterns and flexibility of lower back and hamstrings. However, the simultaneous treatment of the quality of movement patterns and flexibility did not constitute a reliable multi-factor predictive model.

## Conclusions

The risk of an injury increases with low-quality movement patterns and a low level of flexibility of the lower back and hamstrings. Prior injuries increase the risk of further injuries and full recovery is important. Exercises that improve the flexibility and quality of movement patterns and promote the full healing of injuries as preventive measures may effectively reduce the risk of injury. However, further investigation is nedeed. The quality of movement patterns is an accurate predictor for the risk of injury and is applicable here. Flexibility is a weak predictor after six months of observation and should not be used as a predictive tool.

##  Supplemental Information

10.7717/peerj.11399/supp-1 Supplemental Information 1 An empty questionnaire used in the original language

10.7717/peerj.11399/supp-2 Supplemental Information 2 Injury History Questionnaire Completed by all participating subjects with appropriate information about their injury.

10.7717/peerj.11399/supp-3 Supplemental Information 3 Raw data Morphological measurements, FMS test scores, sit-and-reach test scores, and a number of injuries.

## Additional Information and Declarations

### Data Availability

The following information was supplied regarding data availability: The raw measurements are available in the Supplementary File.
