# Supplementary figures and images for: Prediction and injury risk based on movement patterns and flexibility in a 6-month prospective study among physically active adults
Source: PeerJ. 2021 May 18;9:e11399. doi: 10.7717/peerj.11399 (PMC8139277; doi:10.7717/peerj.11399)

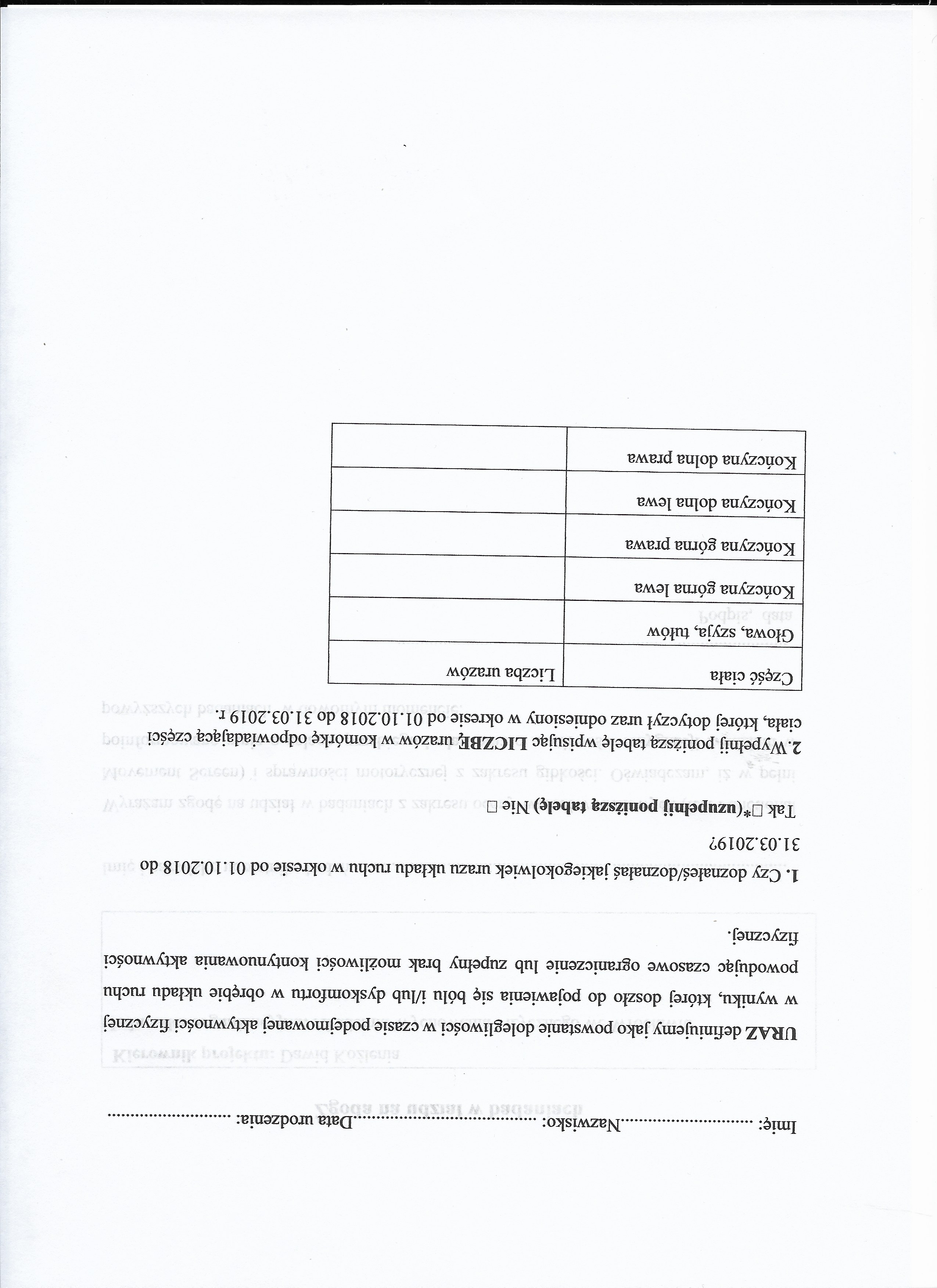

Supplement: Supplemental Information 1 [file peerj-09-11399-s001.pdf]
