# Supplementary material for: Prediction and injury risk based on movement patterns and flexibility in a 6-month prospective study among physically active adults
Source: PeerJ. 2021 May 18;9:e11399. doi: 10.7717/peerj.11399 (PMC8139277; doi:10.7717/peerj.11399)
Supplement: Supplemental Information 2 — Completed by all participating subjects with appropriate information about their injury. [file peerj-09-11399-s002.docx]

**Injury History Questionnaire**

Name: .............................Surname: ........................................Birth date: ...........................

**Injury definition -** In these studies, the injury was defined as the occurrence of complaints during physical activity, which resulted in pain and discomfort in the locomotor system, causing temporary limitation or complete inability to continue physical activity.

**1.** Have you suffered any motor system injury between 01/10/2018 and 31/03/2019?

YES*(  **complete the table below)** NO 

**2**. Fill in the table below by entering the **NUMBER** of injuries in the cell corresponding to the part of the body that was injured during the period from 01/10/2018 to 31/03/2019

| Body part | Injury number |
| --- | --- |
| Haed, neck, torso |  |
| Upper limb -left |  |
| Upper limb - right |  |
| Lower limb - left |  |
| Lower limb - right |  |
